# Concurrent Validity of BrainCheck Compared to SLUMS in Older Adults Using Real-World Clinical Data

**DOI:** 10.1093/geroni/igaf122.4090

**Published:** 2025-12-31

**Authors:** Duong Huynh, Mary Patterson, Bin Huang

**Affiliations:** BrainCheck Inc., Austin, Texas, United States; BrainCheck Inc., Austin, Texas, United States; BrainCheck Inc, Austin, Texas, United States

## Abstract

This retrospective study evaluated the psychometric and diagnostic properties of BrainCheck Assess (BC-Assess), a digital cognitive testing tool, compared with the Saint Louis University Mental Status (SLUMS) exam, a widely used paper-based screener. Real-world clinical data collected across 22 clinics from 2,038 individuals aged 50 years or older who completed both instruments on the same day were analyzed. Participants were classified into Normal (N = 354), Mild Cognitive Impairment (MCI; N = 759), and Dementia (N = 925) groups based on SLUMS criteria. The sample was 56% female, 94% had at least a high school education, and the mean (SD) age was 73.6 (9.3) years. Overall scores on BC-Assess and SLUMS were strongly correlated (r = 0.75), indicating substantial convergence in measuring global cognitive function. Subscore-level analyses revealed moderate correlations between conceptually matched tasks. Canonical correlation analysis identified two major shared cognitive dimensions: the first reflected the role of effective encoding, maintenance, and retrieval of information, and the second captured the coordinated engagement of working memory, attentional control, and executive functioning essential for adaptive cognitive regulation. ROC analyses using SLUMS diagnostic categories as reference showed good discriminative ability for BC-Assess, with AUC values of 0.80 (Normal vs. MCI+Dementia) and 0.84 (Normal+MCI vs. Dementia). Optimal BrainCheck cutoff scores demonstrated balanced positive and negative percent agreement (0.71–0.79) across cutoff determination and validation sets, highlighting strong generalizability. These results support BC-Assess as a valid digital screening tool aligned with SLUMS, providing enhanced domain-specific cognitive insights for clinical and research use.

